# Don't forget your hammer if you want to go to work!

**DOI:** 10.1530/EDM-13-0034

**Published:** 2013-08-30

**Authors:** Mohd Shazli Draman, Aoife Brennan, Michael Cullen, John Nolan

**Affiliations:** 1 Department of Diabetes and EndocrinologySt James's HospitalJames's Street, Dublin 8Ireland

## Abstract

Bilateral lower limb paraesthesia is a common diabetic neuropathy presentation in any busy diabetic clinics. We present a case of a 28-year-old man with a long history of type 1 diabetes mellitus presented with bilateral paraesthesia of both feet and unsteady gait. The patient was able to feel a 10 g monofilament. The presence of brisk reflexes and upgoing plantars in this patient were pointers that further evaluation was warranted. Further investigations revealed diagnosis of subacute combined degeneration of spinal cord. The patient had rapid symptomatic improvement with i.m. vitamin B12 injection. The high volume of patients attending the outpatients with diabetes and paraesthesia can blind us to other possible diagnoses. This article emphasizes that peripheral neuropathy in a diabetic may be due to aetiologies other than diabetes.

**Learning points:**

- Pernicious anaemia is known to be more common in patients with type 1 diabetes.
- Cobalamin deficiency is reversible if detected early enough and treated by B12 replacement.
- By contrast, diabetic neuropathy is generally a progressive complication of diabetes.
- Peripheral neuropathy in a diabetic may be due to aetiologies other than diabetes.

## Background

The term subacute combined degeneration of the spinal cord in vitamin B12 deficiency was introduced by Russell *et al*. in 1900 but the commonest and earliest neurological involvement is a peripheral neuropathy (1). Cerebellar dysfunction and cranial neuropathies have been rarely reported (2). The pathology of peripheral nerve involvement is not absolutely clear whereby both demyelination and axonal loss have been described. We report a case of a patient with type 1 diabetes mellitus who presented with features of subacute combined degeneration of the cord. This article emphasizes that peripheral neuropathy in a diabetic may be due to aetiologies other than diabetes.

## Case presentation

A 28-year-old man with an 8-year history of type 1 diabetes mellitus presented to our Diabetic Day Centre with a 2-month history of paraesthesia of both feet. He had been a poor attendee at the service and had been known to have suboptimal glycaemic control (HbA1c 8.3%). Initially, the diagnosis was thought to be diabetic neuropathy. On further questioning, however, he also reported unsteadiness of gait. He had had several falls particularly in the shower while washing. He had no visual disturbance or symptoms relating to his upper limbs. Apart from diabetes, he had no other medical history and his only treatment was basal-bolus insulin. He was a non-smoker and consumed <10 units of alcohol per week. He was employed as a warehouse attendant. He had no family history of endocrinopathy.

On examination, he had brisk reflexes in both lower limbs with associated clonus. Tone, power and reflexes were normal in both upper limbs. Sensory examination revealed decreased vibration and proprioception to the sternum. Of note, he was able to feel a 10 g monofilament on both feet. Romberg's sign was positive and there were no cerebellar or cranial nerve findings.

## Investigation

This patient had normal haemoglobin of 15.4 g/dl (13.0–18.0) with high mean corpuscular volume of 112fl (80–97) and mean corpuscular haemoglobin of 40.5 pg (27.0–32.0). His blood film showed macrocytes. Routine biochemistry, folate, thyroid function test, syphilis serology, and vasculitic screen were normal. B12 level was reduced on two occasions: 107 and 120 ng/l (150–1000). Parietal cell antibodies were positive. Intrinsic factor antibodies were negative. His MRI brain and spine were normal apart from minimal degenerative changes. Lumbar puncture revealed no cells with normal protein and glucose. Cerebrospinal fluid electrophoresis did not reveal any oligoclonal bands. Nerve conduction studies raised the possibility; the site of abnormality is in the region of cervicomedullary junction and upper cervical region with slowing of large sensory fibres. Gastroscopy with gastric biopsies was consistent with chronic atrophic gastritis and duodenal biopsies were normal. Our investigations confirmed our clinical suspicion of subacute combined degeneration of the cord.

## Treatment

The patient was commenced on a course of i.m. B12 replacement initially on alternate days for 3 weeks followed by maintenance every 2 months.

## Outcome and follow-up

Following the treatment, patient had a rapid symptomatic improvement. He was able to resume work within 2 weeks of starting treatment. The pain and paraesthesia were reportedly much better.

## Discussion

Cobalamin or vitamin B12 deficiency has been recognised as a cause of megaloblastic anaemia for over 100 years. Pernicious anaemia (PA) was originally described by Thomas Addison in 1849 and was later linked to the stomach by Austin Flint. The underlying pathological lesion is the autoimmune destruction of the gastric parietal cells with antibody formation to the parietal cell itself and to its secretory product, intrinsic factor. Intrinsic factor avidly binds to dietary vitamin B12 in the stomach. The B12/intrinsic factor complexes travel to the terminal ileum where they bind to intrinsic factor receptors and are absorbed. The cause of B12 deficiency in the condition is twofold: the destruction of the parietal cell and the presence of the blocking antibodies that prevent B12 and intrinsic factor binding (3).

Neurological abnormalities in PA were originally confused with those of tabes dorsalis. It was in 1892 that the combination of PA and degeneration of the posterior and lateral columns was described. Neurological manifestations are caused by a defect in myelin formation of unknown mechanism. Subacute combined degeneration of the cord describes the degeneration of the dorsal and lateral columns that is virtually exclusively seen in this condition. This initially causes paraesthesia, followed by ataxia and eventually leads to spasticity and paraplegia (4).

PA is known to be more common in patients with type 1 diabetes mellitus. Approximately 15–20% of patients with type 1 diabetes will have positive parietal cell antibodies; these patients have been shown to have an odds ratio of 4.6 for the development of PA (5).

Cobalamin deficiency is reversible if detected early enough and treated by B12 replacement. This is in contrast to diabetic neuropathy, which is generally a progressive complication of the disease albeit slowed by good glycaemic control. Some screening tests for neuropathy currently in use will not detect subacute combined degeneration of the cord. Our patient was able to feel a 10 g monofilament when tested in the clinic. The high volume of patients attending the outpatients with diabetes and paraesthesia can blind us to other possible diagnoses. The presence of brisk reflexes and upgoing plantars in this patient were pointers that further evaluation was warranted.

Subacute combined degeneration of the cord is an important cause of numbness and paraesthesia that is reversible if an early diagnosis is made. We would suggest that the tendon hammer and tuning fork not be forgotten in the evaluation of patients with diabetes.

## Patient consent

Written informed consent was obtained from the patient for publication of this case report.

## Author contribution statement

M S Draman and A Brennan were involved in drafting of the manuscript; M Cullen and J Nolan were involved in critical revision of the manuscript; and M S Draman, A Brennan, M Cullen, and J Nolan were the patient's physicians.
